# Mulberry Transcription Factor MnDREB4A Confers Tolerance to Multiple Abiotic Stresses in Transgenic Tobacco

**DOI:** 10.1371/journal.pone.0145619

**Published:** 2015-12-22

**Authors:** Xue-Qin Liu, Chang-Ying Liu, Qing Guo, Meng Zhang, Bo-Ning Cao, Zhong-Huai Xiang, Ai-Chun Zhao

**Affiliations:** State Key Laboratory of Silkworm Genome Biology, Key Laboratory for Sericulture Functional Genomics and Biotechnology of Agricultural Ministry, Southwest University, Chongqing, 400716, China; Chinese Academy of Sciences, CHINA

## Abstract

The dehydration responsive element binding (DREB) transcription factors have been reported to be involved in stress responses. Most studies have focused on *DREB* genes in subgroups A-1 and A-2 in herbaceous plants, but there have been few reports on the functions of DREBs from the A-3–A-6 subgroups and in woody plants. Moreover, mulberry trees are ecologically and economically important perennial woody plants, but there has been little research on its stress physiology, biochemistry and molecular biology. In this study, a *DREB* gene from the mulberry tree, designated as *MnDREB4A*, classified into the A-4 subgroup by our previous study, was selected for further characterization. Our results showed that the MnDREB4A protein was localized to the nucleus where it activated transcription. The promoter of *MnDREB4A* can direct prominent expression downstream of the β-glucuronidase (GUS) gene under heat, cold, drought and salt stress, and GUS staining was deepest after 12 h of stress treatment. The *MnDREB4A*-overexpression transgenic tobacco showed the improved growth phenotype under untreated conditions, such as greener leaves, longer roots, and lower water loss and senescence rates. Overexpression of *MnDREB4A* in tobacco can significantly enhance tolerance to heat, cold, drought, and salt stresses in transgenic plants. The leaf discs and seedlings of transgenic plants reduced leaf wilting and senescence rates compared to the wild type plants under the different stress conditions. Further investigation showed that transgenic plants also had higher water contents and proline contents, and lower malondialdehyde contents under untreated condition and stress conditions. Our results indicate that the MnDREB4A protein plays an important role in plant stress tolerance.

## Introduction

Abiotic stresses, such as heat, cold, high-salt levels, and drought, present major challenges to sustainable food production because they reduce potential yields by up to 50% in crop plants [1] Therefore, developing crops that are better adapted to abiotic stresses is important for food production in many parts of the world. Major efforts have been made to enhance stress-tolerance through the use of conventional plant breeding, and generated many crop varieties with improved stress tolerance. In this procedure, the stress resistant genetic variability is identified by screening germplasm collections and selections. Beneficial traits are subsequently introduced into cultivars using complex mating designs [2]. However, this approach is time-consuming, and labor- and cost-intensive. Furthermore, unwanted, linked traits can also be transferred, which means that the method can suffer from poor selectivity [3]. Contrary to the conventional plant breeding methods, genetic engineering can directly introduce specified genes into plants, which offers the possibility of improving stress tolerance by a more attractive and quick method [4,5]. To date, genetic engineering has been successfully used to improve plant yield and quality [6], increase pest resistance [7], reduce weed competition [8] and increase abiotic stress tolerance [9], and it has been widely applied to many different species, such as rice [9,10], wheat [8], maize [11], potato, [12] and tomato [13]. For abiotic stress, the inducible genes are classified into functional and regulatory genes according to the functions of their encoding proteins [14]. Introducing a single, functional gene has not been always successful in conferring tolerance because there are many different pathways involved in controlling plant abiotic stress. Conversely, the expression of a regulatory gene, especially a stress related transcription factor, can improve plant abiotic stress tolerance more effectively [15] because it is expected to modulate the expression of a large number of relevant downstream genes [16]. Thus, transcription factor genes will probably become a powerful tool in the genetic engineering of plant abiotic stress tolerance [17,18].

Recently, researchers have identified several key transcription factors (TFs) involved in the regulation networks activated during abiotic stress [19,20], including ethylene responsive element binding factors (ERF), basic-domain leucine-zipper (bZIP), myeloblastosis (MYB), and WRKY binding transcription factors [21,22]. Probably the best studied group of TFs involved in abiotic stress is the dehydration responsive element binding proteins (DREB) genes belonging to the AP2/ERF family. The first DREB family member was isolated from *Arabidopsis* and it responds to low temperature and water deficit [23]. All DREBs contain a conserved AP2 (Apetala2) domain of approximately 60 amino acids and it can bind to the dehydration responsive element (DRE)/c-repeat sequence (CRT) core sequences, which are present in numerous abiotic stress responsive genes promoters [24]. In this domain, there are seven key residues that are essential for highly specific interactions with the DRE/CRT element. There are four args, two trps and one val residues [25]. In the N-terminal of the AP2 domain, the YRG element, containing approximately 20 amino acids, plays an important role in identifying and binding to the DRE/CRT element, especially the Val (V14) at position 14 and Glu (E19) at position 19 [26,27]. The N-terminal of the AP2 domain contains the RAYD element, which has approximately 42 amino acids and the associated WLG motif. Both regulate the special binding activity of TFs by influencing the conformation of the YRG element or by interacting with other proteins [28].

Plant DREB proteins are classified into six subfamilies according to their function and characterization: A-1, A-2, A-3, A-4, A-5, and A-6 [26]. Many of them have been identified and functionally characterized in different species. Most studies have focused on the *DREB* genes in the A-1 and A-2 subgroups, overexpression of A-1 and A-2 families *DREB* genes can induce the expression of stress responsive downstream genes and enhance abiotic stress tolerance [29–35]. However, there have been few reports on the functions of DREBs from the A-3~A-6 subgroups.

Mulberry (*Morus alba* L.) trees are ecologically and economically important perennial woody plants [36]. It had been reported that mulberry can adapt to many different situations, including cold, waterlogged, drought and saline environments [37]. However, there has been little research on its stress physiology, biochemistry and molecular biology [38], and there is very little information available about the mulberry DBRE functions. In our previous study, we identified and characterized 30 DREB genes from the *M*. *notabilis* Genome Database [39], and their functions during abiotic stress were preliminarily predicted.

Based on our previous research, *MnDREB4A* was classified into A-4 subgroup and it can be induced by multiple abiotic stresses [39]. Furthermore, the promoter of this gene contained several *cis*-elements that were related to adverse stresses. Therefore, we selected *MnDREB4A* for further investigation. In this study, we characterized its subcellular localization, promoter activity and function. The tolerance of the transgenic tobacco harboring *MnDREB4A* to multiple abiotic stresses was also evaluated.

## Materials and Methods

### Cloning and bioinformation analysis of the *MnDREB4A* gene

The coding sequence (CDS) of the *MnDREB4A* was amplified and cloned form *M*. *notabilis* as described in our previous study [39]. All of the logged DREB protein sequences for other species were downloaded from the following websites: the National Center for Biotechnology Information (NCBI, http://www.ncbi.nlm.nih.gov/); the *Arabidopsis* information resource (TAIR, http://www.arabidopsis.org/); EnsemblPlants (http://plants.ensembl.org/index.html); the database of rice transcription factors (DRTF, http://drtf.cbi.pku.edu.cn/index.php) and the plant transcription factor database (Plant TFDB, http://planttfdb.cbi.pku.edu.cn/). The information about these sequences is shown in S1 Table. The sequences were aligned using Clustal X 2.0 [40] and illustrated using the Genedoc program [41]. The conserved motifs in MnDREB4A proteins were identified using a motif based sequence analysis tool called MEME [42]. The three-dimensional structure of the MnDREB4A protein was predicted using SWISS-MODEL (http://swissmodel.expasy.org/). The subcellular localization of MnDREB4A proteins was predicted using two website tools: TargetP 1.1 Server (http://www.cbs.dtu.dk/services/TargetP/) and Euk-mPLoc 2.0 (http://www.csbio.sjtu.edu.cn/bioinf/euk-multi-2/).

### Subcellular localization of the MnDREB4A protein

*MnDREB4A* was combined with the N-terminal or C-terminal of green fluorescent protein (EGFP) to construct a fusion protein expression cassette. The *MnDREB4A* and *EGFP* were inserted into the *Kpn* I and *Eco*R I site of the plant expression vector pLGNL, which had been preserved in our laboratory. The gene specific primers that contained restriction enzyme sites are shown in S2 Table. The vectors that contained one of the *MnDREB4A*::*EGFP* fusion, *EGFP*::*MnDREB4A* fusion genes or the *EGFP* expression cassette were confirmed by sequencing and then transiently introduced into onion (*Allium cepa* L.) epidermal cells by *Agrobacterium tumefaciens* LBA4404, as described by Liu *et al*. [43]. The nuclei were stained by DAPI (4', 6-diamidino-2-phenylindole) and images were captured for each color channel with a fluorescence microscope (Nikon, Tokyo, Japan).

### Construction of the MnDREB4A promoter-GUS fusion expression plasmid and *Arabidopsis* transformation

The 1500 bp 5′ upstream region of *MnDREB4A* and the GUS sequence were amplified by PCR using the specific primers with restriction enzyme sites shown in S2 Table. The promoters were fused with the GUS gene and then the fusion construct was inserted into the *Kpn* I and *Eco*R I sites of the pCAMBIA2300 vector. The reconstructed plasmid (*MnDREB4A pro*::*GUS*) was confirmed by sequencing and transformed into *Agrobacterium tumefaciens* EHA 105 by the freeze—thaw method [44]. The positive *Agrobacterium tumefaciens* was transformed into *Arabidopsis thaliana* by the floral dip method [45]. Transgenic *Arabidopsis* were selected on 1/2 MS medium containing a final concentration of 50 mg/L kanamycin and confirmed by PCR analysis. The plasmid *MnDREB4A pro*::*GUS* was used as a positive control and the genomic DNA of wild type (WT) *Arabidopsis* was used as negative control in the PCR analysis.

### Southern blotting and GUS staining of transgenic *Arabidopsis*

Genomic DNAs from the WT and the different transgenic *Arabidopsis* lines were prepared from leaves using the cetyltrimethyl ammonium bromide method (CTAB) [46]. Exactly 20 μg of DNA per sample was digested with *Eco*R I overnight at 37°C. The WT *Arabidopsis* genomic DNA was used as the negative control. The digested DNAs were separated on 0.8% (w/v) agarose gel using electrophoresis and then transferred to a positively charged Hybond-N membrane (Roche Diagnostics GmbH, Mannheim, Germany). The *GUS* probe, labeled with digoxigenin (DIG), was synthesized by PCR followed the manufacturer’s instructions (Roche Diagnostics GmbH, Mannheim, Germany). The Southern blot was finally performed following the pre-hybridization, washing and chemiluminescent detection procedure [47].

GUS activity was assessed histochemically using kanamycin-resistant T1 and T2 generation transgenic *Arabidopsis* that contained *MnDEEB4A pro*::*GUS*. The staining solution for the GUS assay was prepared as described by Rae *et al*. [48]. The plants were incubated with the staining solution at 37°C for about 12 h. The 7-day-old seedlings in each sample were exposed to 40°C (heat), 4°C (cold), 150 mM NaCl or 20% PEG6000 (drought) treatment. The samples were subjected to GUS staining after 0, 1, 3, 6, 9, 12, and 24 h of exposure. The GUS staining was quantified by ImageJ (National Institutes of Health, Maryland, USA). All results are representative of three independent experiments.

### Construction and transformation of the vector for tobacco transformation

The *MnDREB4A* sequence, flanked by a *Kpn* I and a *Spe* I restriction site, were amplified by PCR using the specific primers shown in S2 Table. The obtained DNA fragments were digested by corresponding restriction enzymes and then inserted between the *Kpn* I and *Spe* I sites of the plant expression vector pLGNL under the control of the cauliflower mosaic virus (CaMV) 35S promoter. The recombinant plasmid (*CaMV35S*::*MnDREB4A*) was confirmed by sequencing and transformed into *Agrobacterium tumefaciens* EHA 105 using the freeze—thaw method [44]. Then the positive *Agrobacterium tumefaciens* was transformed into tobacco (*Nicotiana tabacum* L.) plants using the leaf disc method [49]. Transgenic tobacco was selected for on 1/2 MS medium containing a final concentration of 50 mg/L kanamycin and confirmed by GUS staining, and genomic PCR. To determine the expression levels of the *MnDREB4A* in transgenic tobacco plants, total RNA was extracted with RNAiso plus (Takara, Dalian, China), the first strand cDNA was synthesized using the Perfect Real Time version of the PrimerScript^™^ RT reagent Kit with gDNA Eraser (Perfect Real Time) (TaKaRa, Dalian, China). The qRT-PCR procedure was the same as the one described in our previous study [38]. The *NtActin* gene (GenBank Accession number: U60489) was used as an internal reference, the forward and reverse primers for amplification were *NtActin* -F (5'- TCACAGAAGCTCCTCCTAATCCA -3') and *NtActin* -R (5'-GAGGGAAAGAACAGCCTGAATG-3'). Besides, the seeds of the confirmed transgenic lines were cultured in MS medium containing 1 mg/L 6-BA for further research.

### Difference analysis between transgenic lines and WT tobacco under untreated conditions

The leaves (taken from the same position on each sample), roots and water loss rates for the transgenic line and WT tobacco were compared under the same growth conditions. In addition, the leaves were cut into 1 cm diameter discs, which were then cultured with 1/2 MS nutrient solution in a climate chamber (Ningbo Southeast Instrument Corporation, Ningbo, China) (25°C, 16 h day/8 h night). The roots were pulled out and cleaned carefully so that they did not break. To determine the water loss rate, the leaves were weighed (FW) immediately and incubated at 30°C with 40% relative humidity and then weighed at designated times (Wt). The water loss rate was calculated as follows:

Water loss rate = (FW - Wt) / FW×100%.

Each measurement was repeated three times.

### Physiological and abiotic stress tolerance analysis of transgenic tobaccos

The transgenic lines and WT tobacco samples were grown on MS medium until root formation and then transferred into soil in a climate chamber (25°C, 16 h day/8 h night). After about 8 weeks, the tobacco plants were approximately 15 cm high. Then they were subjected to heat (40°C), cold (4°C), drought (20% PEG6000), and salt (400 mM NaCl) stresses until significant differences appeared between the transgenic line and WT tobacco. When this occurred, the leaves from the same positions on the sample were collected for relative water content (RWC), free proline and malonaldehyde (MDA) content measurements. Each treatment was replicated three times. To explore the effects of the various treatments on leaves, 1 cm diameter leaf discs were cut from healthy and fully expanded leaves of transgenic and WT tobacco plants. Then the leaf discs were treated with heat, cold, drought, and salt stresses, respectively.

After the abiotic stress treatments, the fresh weights (FW) of the transgenic line and WT tobacco leaves were immediately recorded. The leaves were soaked for 12 h in distilled water at room temperature under a constant light and the turgid weight (TW) was recorded. Total dry weight (DW) was recorded after drying for 24 h at 80°C. RWC was calculated as follows:

RWC (%) = [(FW - DW) / (TW -DW)]×100%

[50].

For the proline content analysis, exactly ~0.5 g of fresh leaves was homogenized in 10 ml of 3% sulfosalicylic acid and centrifuged at 2,000×g for 15 min. The resulting 2 ml extract was incubated with 2 ml of ninhydrin reagent, which contained 2.5% (w/v) ninhydrin, 60% (v/v) glacial acetic acid and 40% 6 M phosphoric acid, and 2 ml of glacial acetic acid, at 100°C for 40 min. The reaction was terminated in an ice bath and 5 ml of toluene was added. The samples were then vortexed. The reaction mixture was incubated at 23°C for 24 h and the absorbance of the supernatant at 520 nm was determined by a spectrometer (TECHCOMP, Shanghai, China). Then the proline content was calculated as described previously [51].

The MDA content was determined as described by Hodges *et al*. [52]. Around 0.5 g of fresh leaves was homogenized in 10 ml 10% trichloroacetic acid (TCA) and centrifuged at 12,000×*g* for 10 min. Then 2 ml 0.6% thiobarbituric acid (TBA) dissolved in 10% TCA was added to a 2 ml aliquot of the supernatant. The mixture was heated in boiling water for 30 min and then quickly cooled in an ice bath. After centrifugation at 2,500×*g* for 10 min, the absorbance of the supernatant at 450, 532, and 600 nm was determined with a spectrometer (TECHCOMP, Shanghai, China). Then the MDA content was calculated as described previously [52].

### Statistical analysis

The results were organized and analyzed by Excel 2013 (Microsoft, Redmond, USA), Origin 7.0 (OriginLab, MA, USA) and SPSS Statistics 17.0 (SPSS Inc., Chicago, IL, USA) software. The means were compared by Student’s t-test were calculated to find the significant difference between different lines. Mean values that were significantly different within treatment from each other were indicated by asterisks.

## Results

### Bioinformation analysis of the *MnDREB4A* gene

**Fig 1:**
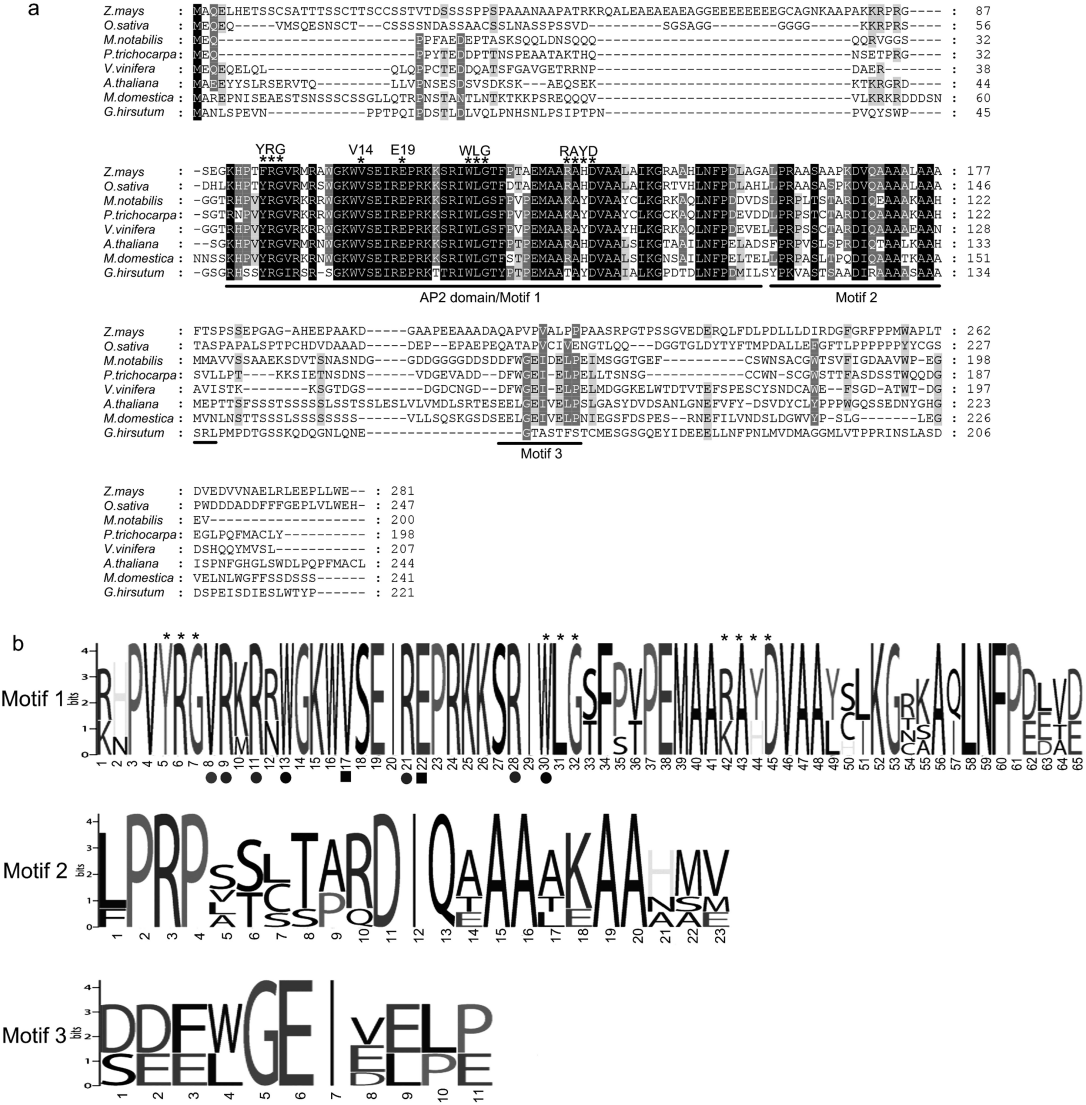
Protein sequence multi-alignment and sequences logos for DREBs from different species. (a) Alignment was performed using the GeneDoc program. The conserved V14, E19, YRG, RAYD and WLG motifs are highlighted by asterisks and lines. Conservative sequences are highlighted by black and grey shading. (b) The overall height of each stack indicates the conservation of the protein sequence at the amino acid position, whereas the heights of the letters within each stack represent the relative frequency of the corresponding amino acid. The YRG, RAYD and WLG motifs are highlighted by asterisks. The key residues that are essential for the highly specific interactions with and for binding to the DRE/CRT element are highlighted by colorful circles and squares.

Multiple sequence analysis showed that all deduced proteins exhibited the typical features of DREB proteins, including an AP2 domain, and YRG, WLG, and RAYD motifs (Fig 1a). Moreover, these proteins also had the conserved valine (V14) at the 14th position and glutamic acid (E19) at the 19th position in the AP2 domain. The AP2 domain displayed a higher degree of amino acid identity than the N-termini and C-termini of the proteins.

To gain insight into the nature of the AP2 domain of mulberry DREB protein and its homologues from other species, including *Populus trichocarpa*, *Vitis vinifera*, *Malus domestic*, *Gossypium hirsutum*, *Arabidposis thaliana*, *Zea mays*, and *Oryza sativa*, sequence logos were produced to examine the level of conservation at each residue position. The results revealed that 46 (70.8%) of the conserved amino acids were identical in the AP2 domain, and the residues located in other positions displayed varying levels of conservation (Fig 1b). In addition to V14 and E19, there were four arginine (R), two tryptophan (W) and a valine (V) participating in the interaction between the DREB proteins and specific DNA sequences (Fig 1b). Apart from the AP2 domain, two other conserved motifs were also found (Fig 1b), but the functions of these motifs require further investigation.

Based on the SWISS-MODEL online prediction, the *MnDREB4A* encoding a protein containing three β-folded sheets and one α-helix (S1a Fig). The α-helix packed approximately parallel to the β-folded sheets. The V14 and E19 residues, which allow the protein to bind DNA, were located in the second β-folded sheet (β-2) (S1b Fig). When the model was rotated at certain angles, the second β-folded sheet (β-2) was in the most favorable position to bind the DNA (S1c Fig), which was consistent with that of other reported *DREB* genes[25].

### Subcellular localization of the MnDREB4A protein

**Fig 2:**
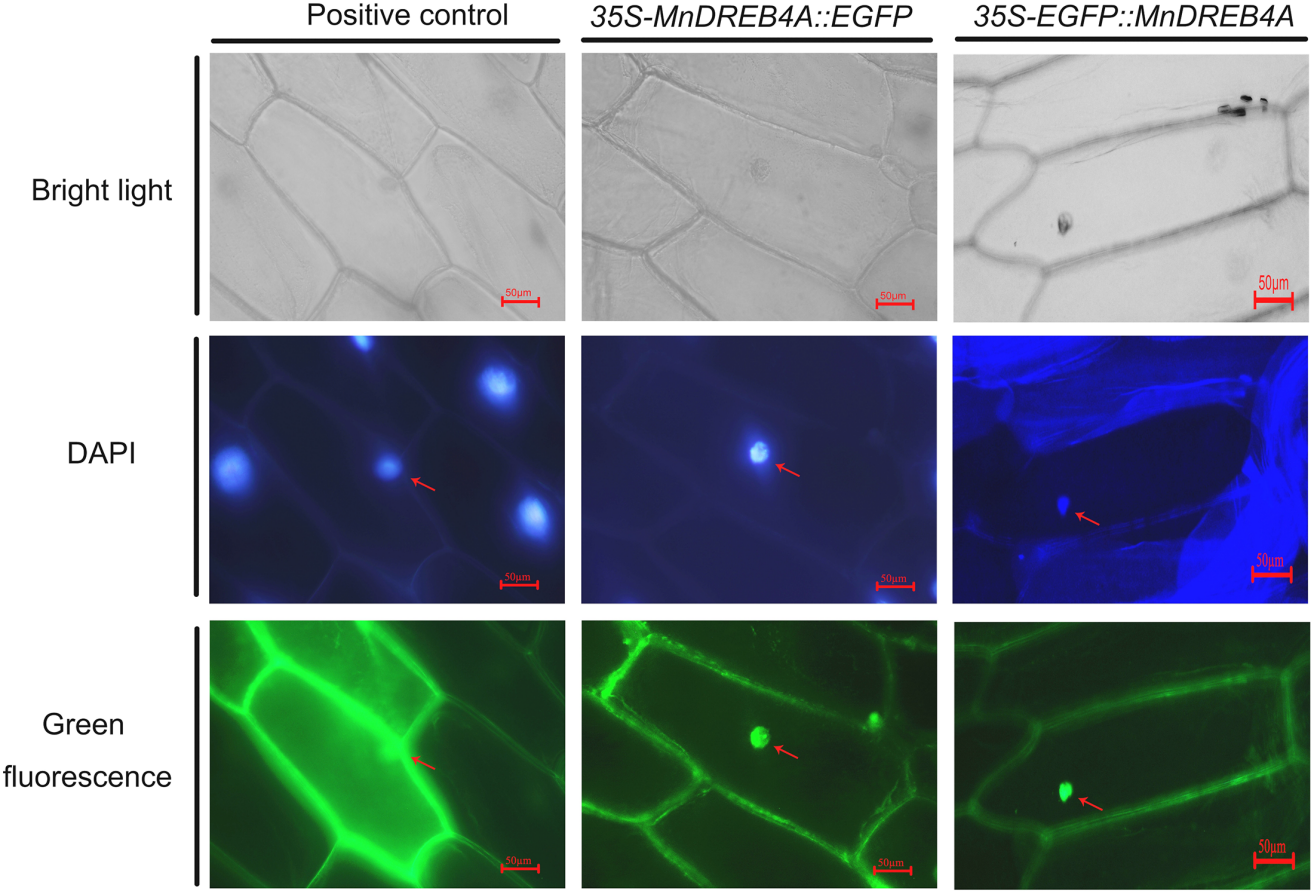
Subcellular localization of the MnDREB4A transcription factor in onion epidermal cells. The *MnDREB4A*::*EGFP* or *EGFP*::*MnDREB4A* fusion genes under the control of CaMV 35S promoter were transfected into onion epidermal cells. The positive control was a transfected plasmid containing *EGFP* alone. DAPI and arrows were used to indicate the nucleus. Images were captured for each color channel with a fluorescence microscope (Nikon, Tokyo, Japan).

Although the MnDREB4A didn’t contain an obvious nuclear localization signal (NLS), it was predicted to be nuclear protein according to the TargetP 1.1 Server and Euk-mPLoc 2.0. To validate the prediction, the *MnDREB4A*::*EGFP* vector and the *EGFP*::*MnDREB4A* fusion gene, under the control of the CaMV 35S promoter (S2 Fig), were transfected into the onion epidermal cells. The *EGFP* vector was used as a control and was transfected separately (S2 Fig). The green fluorescence signals of the MnDREB4A::EGFP and EGFP::MnDREB4A fusion proteins were observed only in the nucleus (Fig 2), which implied that the MnDREB4A proteins were nucleus-localized proteins. This result was consistent with the protein structure prediction.

### Promoter activity analysis

To examine whether the abiotic stress responsiveness of the *MnDREB4A* gene was transcriptionally controlled by 5′ upstream regions, transgenic *Arabidopsis* were generated by introducing a *MnDREB4A pro*::*GUS* fused gene (S3a Fig). Kanamycin-resistant progeny (T1 generation) were confirmed by genomic PCR (S3b Fig). The PCR result showed that the *GUS-nos* and *MnDREB4A pro* genes could be detected in *Arabidopsis* transgenic strains 1, 2, 6, 9 and 10 (designated as TS1, TS2, TS6, TS9, and TS10). The Southern blotting showed that there was only one copy of *MnDREB4A pro*::*GUS* in TS1, TS2, and TS9, and multiple copies in TS6 and TS10 (S3c Fig).

**Fig 3:**
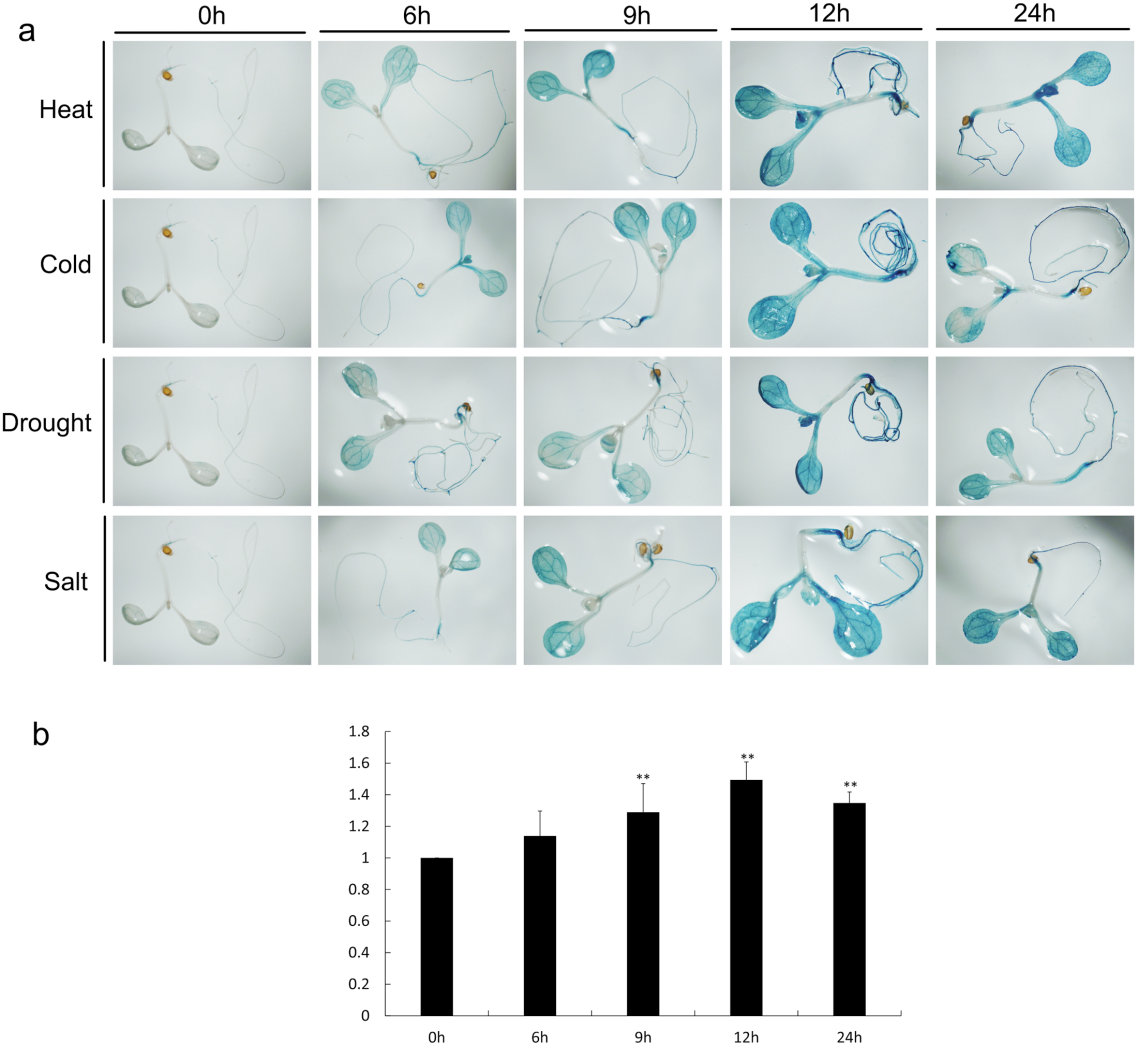
Expression patterns for the *GUS* reporter gene under the control of the *MnDREB4A* promoter in transgenic *Arabidopsis*. (a) Histochemical GUS staining in TS2 was performed after abiotic stress treatment at designated time points. Heat: 40°C, Cold: 4°C, Salt: 150 mM NaCl, Drought: 20% PEG6000. (b) The GUS staining was quantified by ImageJ (National Institutes of Health, Maryland, USA).

One-week-old T2 seedlings were treated with heat (40°C), cold (4°C), drought (20% PEG6000), and salt (150 mM NaCl). After the different treatments, the strongest *GUS* expressions were observed in the leaves and roots of TS2 (Fig 3), TS1, TS9, and TS10 (S4a–S4c Fig), and lower in TS6 (data not shown). The GUS activity was the highest after 12 h treatment and then declined. Although the GUS accumulation levels in the different transgenic lines were different, their response trends under abiotic stresses were identical. We also found that the *GUS* reporter was mainly expressed in the leaves and roots and it preferentially localized to the vasculature of various tissues.

### The difference between transgenic line and WT tobacco under normal conditions

**Fig 4:**
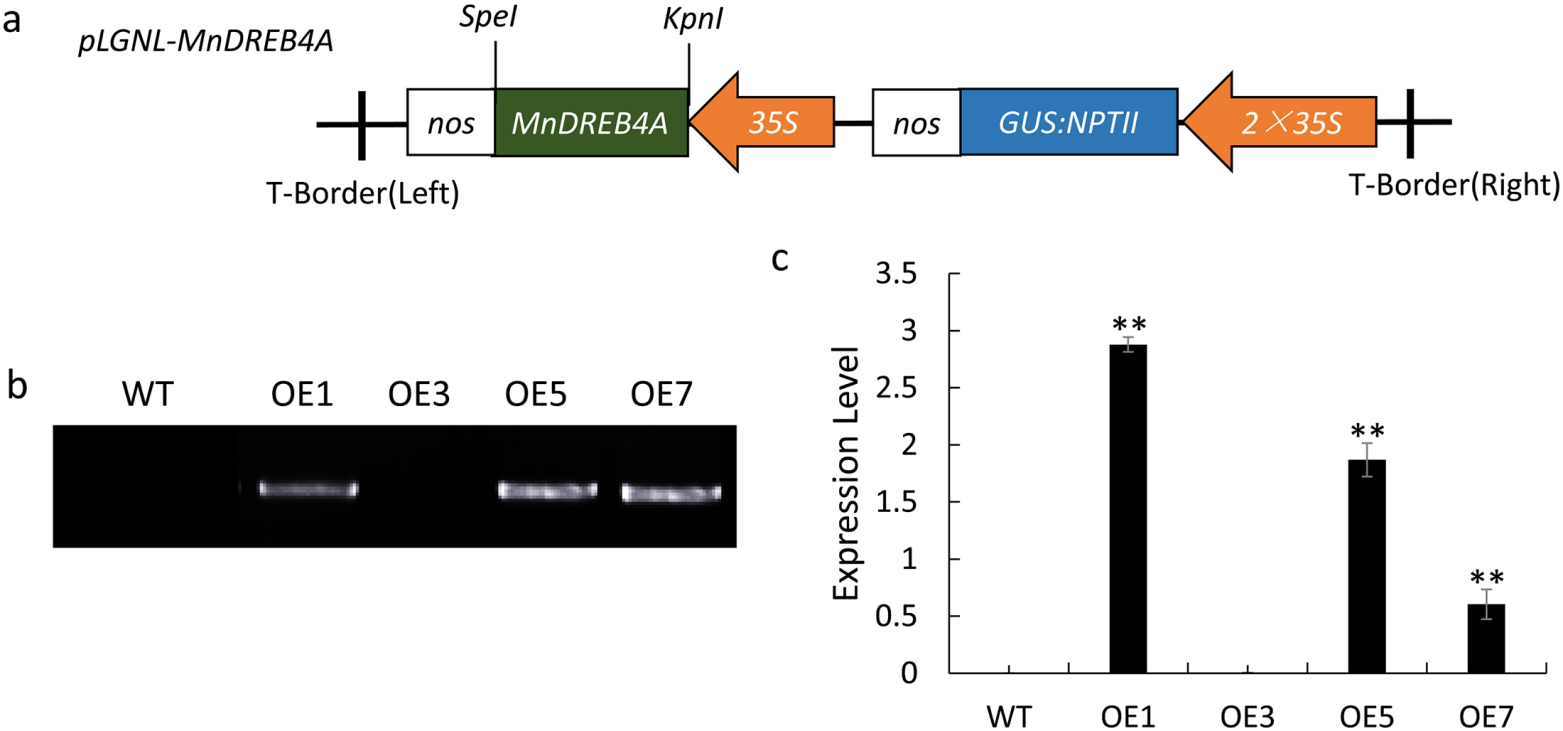
The recombinant plasmid (*CaMV35S*::*MnDREB4A*) used in the transgenic tobacco, and the PCR and qRT-PCR analysis results. (a) The recombinant plasmid (*CaMV35S*::*MnDREB4A*). (b) PCR analysis using genomic DNA as the template, and 35S forward and gene-specific reverse primers. (c) The expression level of the *MnDREB4A* gene in the transgenic lines and the control plants was examined by qRT-PCR analysis. The data were calculated using the 2^–ΔΔCt^ formula. The significantly different values among WT and transgenic tobacco plants are indicated with asterisks (n = 3). Two asterisks indicate a significant difference at *P < 0*.*01*.

To investigate *MnDREB4A* function, the recombinant plasmid (*CaMV35S*::*MnDREB4A*) (Fig 4a) was transformed into tobacco and produced transgenic plants. These transgenic lines were selected on selection medium containing kanamycin. The transgenic plants were analyzed further by PCR using genomic DNA as the template, and 35S forward and gene-specific reverse primers (Fig 4b). The expression levels of the *MnDREB4A* gene in the transgenic lines and the control plants (WT) were examined by qRT-PCR analyses (Fig 4c). Expression of *MnDREB4A* in transgenic lines 1, 5 and 7 (designated as OE1, OE5, and OE7) was over 300-fold greater than in the control plants (Fig 4c). The OE1 transgenic line was chosen for further experiments.

**Fig 5:**
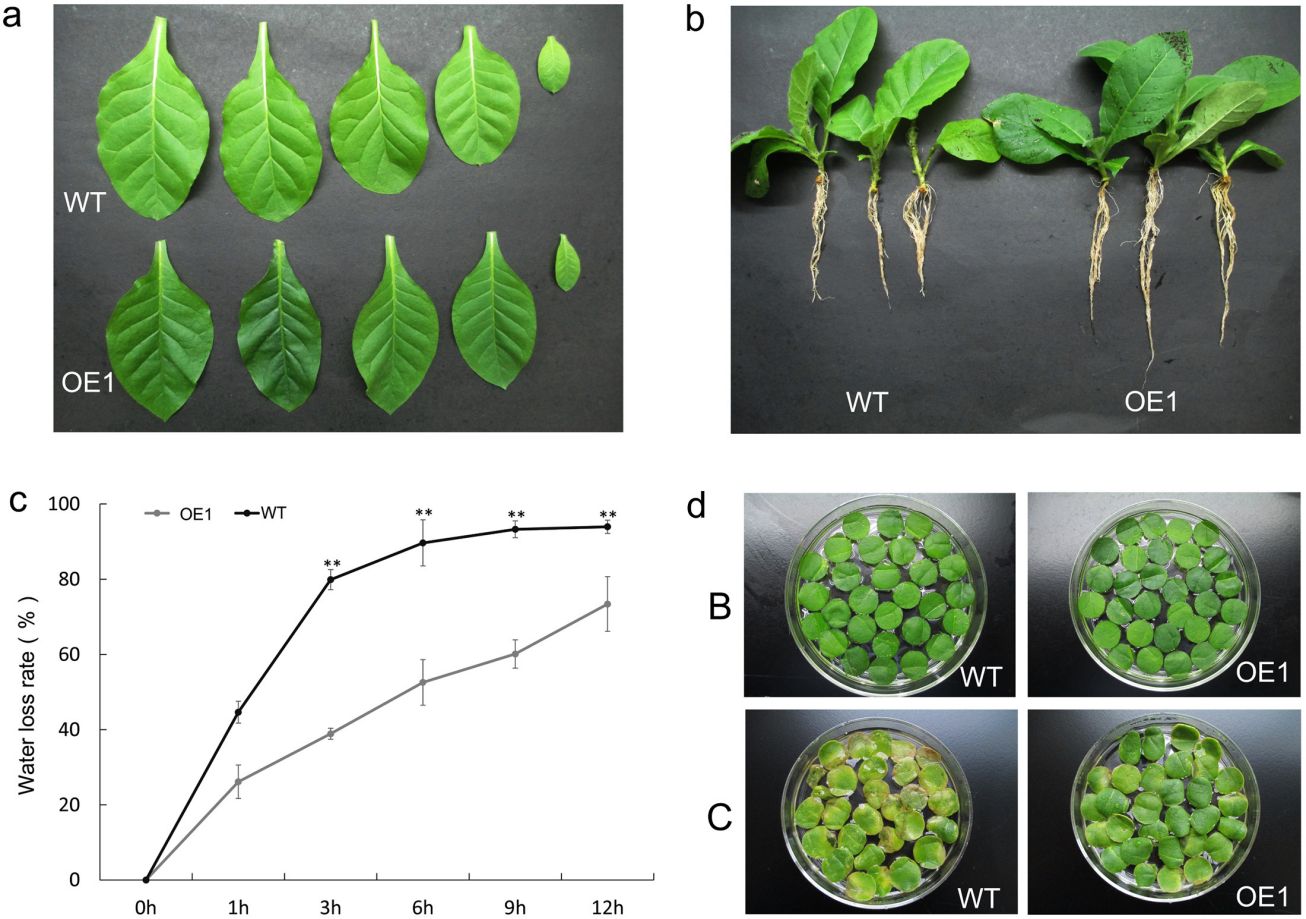
The differences between the transgenic line and WT tobacco under normal conditions. (a) The OE1 leaves were smaller and greener than the WT leaves. (b) The OE1 roots were longer than the WT roots. (c) The OE1 plants showed lower rates of water loss at each time point than the WT after leaf detachment and incubation at 30°C and 40% relative humidity. The significantly different values between the WT and OE1 plants are indicated by asterisks (n = 3). A single asterisk indicates a significant difference at *P < 0*.*05* and two asterisks indicates a significant difference at *P < 0*.*01*. (d) The leaf discs were cultured in 1/2MS nutrient solution for about a month. The discs from WT plants became yellow and senescence was more obvious than on the transgenic tobacco OE1 leaf discs. The top line is the leaf discs before culturing, and the bottom line is the leaf discs after 1 month’s culturing.

The transgenic OE1 tobacco and the WT were cultivated under the same conditions. However, when compared to the WT under normal conditions, the OE1 tobacco plants showed obvious phenotypic leaf and root differences. For example, the OE1 leaves were smaller and greener than the WT leaves (Fig 5a) and the OE1 roots were longer than the WT roots (Fig 5b). Furthermore, the OE1 plants exhibited lower rates of water loss at each time point than the WT after leaf detachment (Fig 5c). When the leaf discs had been cultured in 1/2 MS nutrient solution for about 1 month, the discs from the WT plants became yellow and senescence was more obvious than for the transgenic tobacco OE1 disks (Fig 5d). All these results indicated that the OE1 transgenic tobacco should have a stronger tolerance to adverse conditions than the WT.

### Overexpression of *MnDREB4A* enhanced tolerance to multiple abiotic stresses in transgenic tobacco

**Fig 6:**
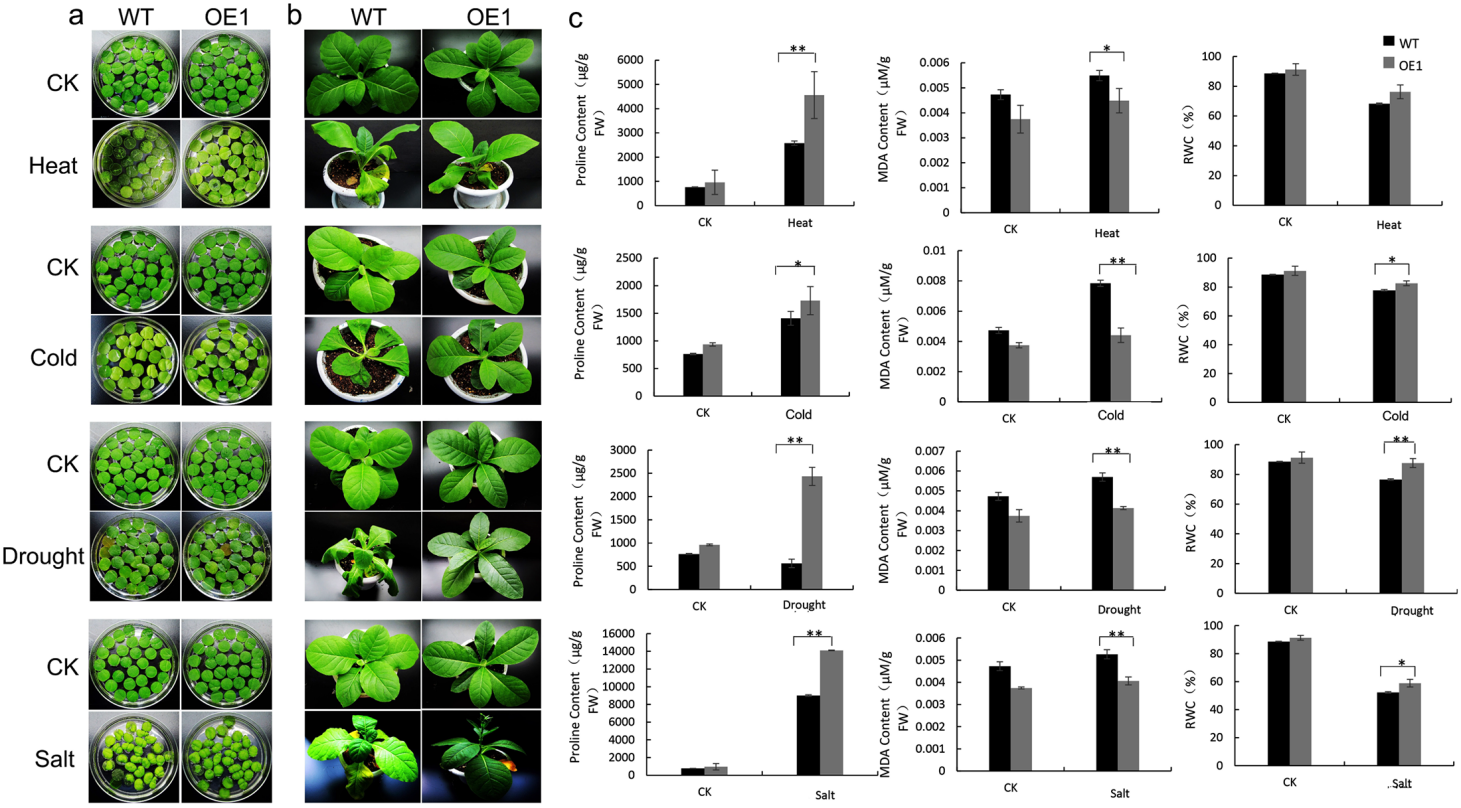
Analysis of enhanced abiotic stress tolerances in the transgenic tobacco line and WT plants. (a, b) The leaf discs or seedlings of transgenic OE1 and the WT (CK) were exposed to heat (40°C), cold (4°C), drought (20% PEG6000) and salt (400 mM NaCl). (c) The proline, MDA content and RWC after the abiotic stresses treatments. The statistically different values between WT and OE1 are indicated by asterisks (n = 3). A single asterisk indicates a significant difference at *P* < 0.05 and two asterisks indicates a significant difference at *P* < 0.01.

The OE1 and WT leaf discs and seedlings were tested with various abiotic stresses to characterize the function of *MnDREB4A* transgenic line under heat, cold, drought, and salt stress. Fig 6a shows that the largest areas of damage were observed in WT tobacco leaf discs after 4 days of heat (40°C) treatment, while OE1 discs suffered only slight damage. After 1 month of cold (4°C) or drought (20% PEG6000) treatment, the WT discs exhibited more obvious chlorosis and senescence than the OE1 discs (Fig 6a). Similarly, the WT discs showed more severe water loss and senescence compared to the OE1 discs after salt (400 mM NaCl) treatment (Fig 6a). When OE1 and WT seedlings were subjected to the treatments, no evident morphological differences were observed between the transgenic OE1 line and the WT seedlings during the first few days of treatment. After heat treatment for 4 days, the WT plants exhibited severe symptoms of water loss and significant wilting, but only slight wilting was observed in some of the OE1 leaves (Fig 6b). When the plants were exposed to 4°C for 6 days, the leaves of the WT seedlings began wilting, whereas the leaves of the OE1 seedlings continued to grow well (Fig 6b), and when the seedlings were treated with 20% PEG6000 (drought) for 8 days, the WT seedlings also showed more obvious leaf wilting and senescence (Fig 6b). After 3 weeks of exposure to 400 mM NaCl, the OE1 seedlings had lower rates of leaf yellowing than the WT plants, and the OE1 plants grew taller and reached the flowering stage sooner than the WT plants (Fig 6b). The phenotype characterization suggested that overexpression of *MnDREB4A* enhanced heat, cold, drought and salt stress tolerance. Some important physiological indices were also measured in order to investigate the physiological differences between OE1 and WT plants. The OE1 transgenic line showed remarkably higher levels of proline (Fig 6c) and increased RWC (Fig 6c) compared to the WT plants, but lower levels of MDA (Fig 6c) under the different abiotic stresses. These results were also confirmed in the OE7 transgenic tobacco (S5 Fig).

In conclusion, the physiological characterization results suggested that the abiotic stress tolerance of transgenic tobacco was considerably enhanced because of expeditious accumulation of stress relieving substances compared to the WT plants.

## Discussion

To survive different abiotic stresses, plants have evolved intricate mechanisms that respond and adapt to these stresses at the molecular, cellular and whole-plant level [1]. Transcription factors (TFs) that regulate signal transduction pathways in response to heat, cold, drought and salt have attracted considerable attention. In our previous study, 30 *DREB* genes were identified in *M*. *notabilis* [39]. The *MnDREB4A* genes were classified into the A-4 subgroup and shown to respond to heat, cold, drought, and salt stresses. In this study, the *MnDREB4A* gene was further characterized to explore the function of this gene in response to various stresses.

Our previous work suggested that the response to different abiotic stresses by *MnDREB4A* may be related to the *cis*-elements in the promoter [39,53]. The promoter analysis showed that the 1500 bp 5’-upstream region of the *MnDREB4A* contained different kinds of *cis*-elements that were related to abiotic stresses (S6 Fig) [43], so we constructed the *MnDREB4A pro*::*GUS* vector and introduced it into *Arabidopsis*. After the stress treatments, the GUS activity of the transgenic *Arabidopsis* seedlings increased and reached its highest level after 12 h treatment (Fig 3 and S4 Fig). This result provided additional evidence that *MnDREB4A* played a role in plant resistance to abiotic stresses.

Studies have demonstrated that overexpression of the stress-inducible TF genes in transgenic plants is an effective strategy for improving abiotic stress tolerance [54]. Transgenic plants were generated in this study that overexpressed the *MnDREB4A* gene in tobacco (Fig 4). However, some reports have found that gene overexpression may cause dwarf plant phenotypes [55], but our *MnDREB4A* transgenic line showed improved growth under normal conditions, such as greener leaves, longer roots, and reduced damage and senescence, etc. (Fig 5). When exposed to abiotic stresses, the transgenic line had lower leaf wilting and senescence rates than the WT plants (Fig 6). These results suggested that overexpression of *MnDREB4A* gene can improve tolerance to abiotic stresses.

To explore the possible mechanisms underlying improved stress tolerance, several experiments were conducted to monitor the changes in physiological processes associated with plant responses to stresses. RWC is often used to measure plant water status under abiotic stresses [56]. In this study, transgenic tobacco had a higher RWC than the WT plants. Plants also accumulate several metabolites that can help to prevent detrimental changes [57]. Proline is a common osmolyte in plants, and proline accumulation is associated with plant responses to stress [58]. Abiotic stress causes lipid peroxidation, which leads to MDA accumulation [59]. Therefore, MDA content could be used as a measure of the damage caused by abiotic stresses [60]. Although physiological status changes are not the only way that plants cope with damage, plants with higher proline and lower MDA contents do show improved stress tolerance [56] and these physiological changes improved transgenic tobacco responses to adverse conditions.

## Conclusion

In conclusion, our results clearly demonstrate that MnDREB4A is a stress inducible transcription factor in mulberry, and *MnDREB4A* can respond to heat, cold, drought and salt stress. Transgenic tobacco overexpressing *MnDREB4A* displayed a higher tolerance to abiotic stresses than the WT plants. Our results suggest that these functions are achieved by altering the plant’s physiological status, such as accumulating water and proline, and reducing plant MDA contents, etc. This is probably because transgenic tobacco can efficiently respond to abiotic stresses. The present study provides some insights into the function of MnDREB4A from the A-4 subgroups of DREB proteins in mulberry plants and lays a foundation for the further understanding of their mechanisms underlying plant responses to abiotic stresses.

## Supporting Information

S1 Fig The three-dimensional structure of MnDREB4A protein predicted by SWISS-MODEL. (a) The protein sequence of MnDREB4A. AP2 domains are highlight by the lines. Three β-folded sheets (β-1, β-2, and β-3) and one α-helix (α) were highlight by colorful arrows and rectangle. (b) The three-dimensional structure of MnDREB4A protein. The V14 and E19 were highlight by triangles. (c) The protein model was rotated a certain angles to show the structure.
(JPG)

S2 Fig The recombinant plasmids for subcellular localization analysis. (JPG)

S3 Fig The recombinant plasmid for promoter activity analysis and the detection of transgenic *Arabidopsis*. (a) The recombinant plasmid (*MnDREB4A pro*::*GUS*). (b) The transgenic lines were confirmed by genomic PCR. (c) Southern blot analysis of transgenic *Arabidopsis*. The recombinant plasmid was used as a positive control, and the genomic DNA of wild type (WT) *Arabidopsis* was used as negative control. P, N, and TSs indicate positive control, negative control, and transgenic lines, respectively.
(JPG)

S4 Fig Expression patterns of *GUS* reporter gene under control of *MnDREB4A* promoter in different transgenic *Arabidopsis* lines. (a) TS1, (b) TS9, (c) TS10.
(JPG)

S5 Fig Analysis of abiotic stress tolerances in the transgenic tobacco line OE7 and WT plants. (a) The seedlings of transgenic OE7 and the WT (CK) were exposed to cold (4°C), heat (40°C), drought (20% PEG6000) and salt (400 mM NaCl). (b) The proline content after the abiotic stresses treatments. (c) The MDA content after the abiotic stresses treatments.
(JPG)

S6 Fig Summary of cis-regulatory elements related to abiotic stresses present in the 5’-upstream region of *MnDREB4A*. The *cis*-elements were highlight by colorful rectangles.
(JPG)

S1 Table The DREB proteins used for protein sequence multi-alignment. (DOCX)

S2 Table List of primers used for constructing recombinant plasmids. (DOCX)

## References

[1] Rashid M, Guangyuan H, Guangxiao Y, Hussain J, Xu Y (2001) AP2/ERF transcription factor in rice: genome-wide canvas and syntenic relationships between monocots and eudicots. Evolutionary Bioinformatics 8: 321–355. 10.4137/EBO.S9369 PMC3396566 22807623

[2] Varshney RK, Bansal KC, Aggarwal PK, Datta SK, Craufurd PQ (2011) Agricultural biotechnology for crop improvement in a variable climate: hope or hype? Trends in Plant Science 16: 363–371. 10.1016/j.tplants.2011.03.004 21497543

[3] Ashraf M, Akram NA (2009) Improving salinity tolerance of plants through conventional breeding and genetic engineering: an analytical comparison. Biotechnology Advances 27: 744–752. 10.1016/j.biotechadv.2009.05.026 19500659

[4] Dunwell JM (2000) Transgenic approaches to crop improvement. Journal of Experimental Botany 51: 487–496. 10938856 10.1093/jexbot/51.suppl_1.487

[5] Wang W, Vinocur B, Altman A (2003) Plant responses to drought, salinity and extreme temperatures: towards genetic engineering for stress tolerance. Planta 218: 1–14. 14513379 10.1007/s00425-003-1105-5

[6] Ye X, Al-Babili S, Klöti A, Zhang J, Lucca P, Beyer P, et al (2000) Engineering the provitamin A (β-carotene) biosynthetic pathway into (carotenoid-free) rice endosperm. Science 287: 303–305. 10634784 10.1126/science.287.5451.303

[7] Fujimoto H, Itoh K, Yamamoto M, Kyozuka J, Shimamoto K (1993) Insect resistant rice generated by introduction of a modified δ-endotoxin gene of *Bacillus thuringiensis* . Nature Biotechnology 11: 1151–1155. 10.1038/nbt1093-1151 7764096

[8] Vasil V, Castillo AM, Fromm ME, Vasil IK (1992) Herbicide resistant fertile transgenic wheat plants obtained by microprojectile bombardment of regenerable embryogenic callus. Nature Biotechnology 10: 667–674.

[9] Oh SJ, Song SI, Kim YS, Jang HJ, Kim SY, Kim M, et al (2005) *Arabidopsis* CBF3/DREB1A and ABF3 in transgenic rice increased tolerance to abiotic stress without stunting growth. Plant Physiology 138: 341–351. 15834008 10.1104/pp.104.059147 PMC1104188

[10] Xu D, Duan X, Wang B, Hong B, Ho T, Wu R. (1996) Expression of a late embryogenesis abundant protein gene, *HVA1*, from barley confers tolerance to water deficit and salt stress in transgenic rice. Plant Physiology 110: 249–257. 12226181 10.1104/pp.110.1.249 PMC157716

[11] Koziel MG, Beland GL, Bowman C, Carozzi NB, Crenshaw R, Crossland L, et al (1993) Field performance of elite transgenic maize plants expressing an insecticidal protein derived from Bacillus thuringiensis. Nature Biotechnology 11: 194–200.

[12] Wu G, Shortt BJ, Lawrence EB, Levine EB, Fitzsimmons KC, Shah DM (1995) Disease resistance conferred by expression of a gene encoding H_2_O_2_-generating glucose oxidase in transgenic potato plants. The Plant Cell Online 7: 1357–1368. 10.1105/tpc.7.9.1357 PMC160957 8589621

[13] Zhang H-X, Blumwald E (2001) Transgenic salt-tolerant tomato plants accumulate salt in foliage but not in fruit. Nature Biotechnology 19: 765–768. 11479571 10.1038/90824

[14] Shinozaki K, Yamaguchi-Shinozaki K (2007) Gene networks involved in drought stress response and tolerance. Journal of Experimental Botany 58: 221–227. 17075077 10.1093/jxb/erl164

[15] Reguera M, Peleg Z, Blumwald E (2012) Targeting metabolic pathways for genetic engineering abiotic stress-tolerance in crops. Biochimica et Biophysica Acta (BBA)-Gene Regulatory Mechanisms 1819: 186–194. 21867784 10.1016/j.bbagrm.2011.08.005

[16] Kizis D, Lumbreras V, Pagès M (2001) Role of AP2/EREBP transcription factors in gene regulation during abiotic stress. FEBS letters 498: 187–189. 11412854 10.1016/s0014-5793(01)02460-7

[17] Agarwal P, Jha B (2010) Transcription factors in plants and ABA dependent and independent abiotic stress signalling. Biologia Plantarum 54: 201–212.

[18] Hussain SS, Kayani MA, Amjad M (2011) Transcription factors as tools to engineer enhanced drought stress tolerance in plants. Biotechnology Progress 27: 297–306. 10.1002/btpr.514 21302367

[19] Seki M, Umezawa T, Urano K, Shinozaki K (2007) Regulatory metabolic networks in drought stress responses. Current Opinion in Plant Biology 10: 296–302. 17468040 10.1016/j.pbi.2007.04.014

[20] Bohnert HJ, Ayoubi P, Borchert C, Bressan RA, Burnap RL, Cushman JC, et al (2001) A genomics approach towards salt stress tolerance. Plant Physiology and Biochemistry 39: 295–311.

[21] Singh KB, Foley RC, Oñate-Sánchez L (2002) Transcription factors in plant defense and stress responses. Current Opinion in Plant Biology 5: 430–436. 12183182 10.1016/s1369-5266(02)00289-3

[22] Huang GT, Ma SL, Bai LP, Zhang L, Ma H, Jia P, et al (2012) Signal transduction during cold, salt, and drought stresses in plants. Molecular Biology Reports 39: 969–987. 10.1007/s11033-011-0823-1 21573796

[23] Stockinger EJ, Gilmour SJ, Thomashow MF (1997) *Arabidopsis thaliana CBF1* encodes an AP2 domain-containing transcriptional activator that binds to the C-repeat/DRE, a cis-acting DNA regulatory element that stimulates transcription in response to low temperature and water deficit. Proceedings of the National Academy of Sciences 94: 1035–1040. 10.1073/pnas.94.3.1035 PMC19635 9023378

[24] Yamaguchi-Shinozaki K, Shinozaki K (1994) A novel cis-acting element in an *Arabidopsis* gene is involved in responsiveness to drought, low-temperature, or high-salt stress. The Plant Cell 6: 251–264. 8148648 10.1105/tpc.6.2.251 PMC160431

[25] Allen MD, Yamasaki K, Ohme-Takagi M, Tateno M, Suzuki M (1998) A novel mode of DNA recognition by a β-sheet revealed by the solution structure of the GCC-box binding domain in complex with DNA. The EMBO Journal 17: 5484–5496. 9736626 10.1093/emboj/17.18.5484 PMC1170874

[26] Sakuma Y, Liu Q, Dubouzet JG, Abe H, Shinozaki K, Yamaguchi-Shinozaki K (2002) DNA-binding specificity of the ERF/AP2 domain of *Arabidopsis* DREBs, transcription factors involved in dehydration-and cold-Inducible gene expression. Biochemical and Biophysical Research Communications 290: 998–1009. 11798174 10.1006/bbrc.2001.6299

[27] Wang ZL, An XM, Li B, Ren YY, Jiang XB, Bo WH, et al (2008) Identification and characterization of CBF/DREB1-related genes in *Populus hopeiensis* . Forestry Studies in China 10: 143–148.

[28] Okamuro JK, Caster B, Villarroel R, Van Montagu M, Jofuku KD (1997) The AP2 domain of APETALA2 defines a large new family of DNA binding proteins in *Arabidopsis* . Proceedings of the National Academy of Sciences 94: 7076–7081. 10.1073/pnas.94.13.7076 PMC21287 9192694

[29] Chen JR, Lü JJ, Wang TX, Chen SY, Wang HF (2009) Activation of a DRE-binding transcription factor from *Medicago truncatula* by deleting a Ser/Thr-rich region. In Vitro Cellular & Developmental Biology-Plant 45: 1–11.

[30] Mizoi J, Ohori T, Moriwaki T, Kidokoro S, Todaka D, Maruyama K, et al (2013) GmDREB2A; 2, a canonical dehydration-responsive element-binding protein2-type transcription factor in soybean, is posttranslationally regulated and mediates dehydration-responsive element-dependent gene expression. Plant Physiology 161: 346–361. 10.1104/pp.112.204875 23151346 PMC3532265

[31] Dubouzet JG, Sakuma Y, Ito Y, Kasuga M, Dubouzet EG, Miura S, et al (2003) *OsDREB* genes in rice, *Oryza sativa* L., encode transcription activators that function in drought-, high-salt- and cold-responsive gene expression. The Plant Journal 33: 751–763. 12609047 10.1046/j.1365-313x.2003.01661.x

[32] Xue GP (2003) The DNA-binding activity of an AP2 transcriptional activator HvCBF2 involved in regulation of low-temperature responsive genes in barley is modulated by temperature. The Plant Journal 33: 373–383. 12535350 10.1046/j.1365-313x.2003.01630.x

[33] Li X, Zhang D, Li H, Wang Y, Zhang Y, Wood AJ (2014) EsDREB2B, a novel truncated DREB2-type transcription factor in the desert legume *Eremosparton songoricum*, enhances tolerance to multiple abiotic stresses in yeast and transgenic tobacco. BMC Plant Biology 14: 44 10.1186/1471-2229-14-44 24506952 PMC3940028

[34] Jin X, Xue Y, Wang R, Xu R, Bian L, Zhu B, et al (2013) Transcription factor *OsAP21* gene increases salt/drought tolerance in transgenic *Arabidopsis thaliana* . Molecular Biology Reports 40: 1743–1752. 10.1007/s11033-012-2228-1 23104474

[35] Bouaziz D, Pirrello J, Charfeddine M, Hammami A, Jbir R, Dhieb A. (2013) Overexpression of StDREB1 transcription factor increases tolerance to salt in transgenic potato plants. Molecular Biotechnology 54: 803–817. 10.1007/s12033-012-9628-2 23250722

[36] Ramachandra Reddy A, Chaitanya K, Jutur P, Sumithra K (2004) Differential antioxidative responses to water stress among five mulberry (Morus alba L.) cultivars. Environmental and Experimental Botany 52: 33–42.

[37] Checker VG, Khurana P (2013) Molecular and functional characterization of mulberry EST encoding remorin (MiREM) involved in abiotic stress. Plant Cell Reports 32: 1729–1741. 10.1007/s00299-013-1483-5 23942844

[38] Wei C, Liu X, Long D, Guo Q, Fang Y, Bian C, et al (2014) Molecular cloning and expression analysis of mulberry *MAPK* gene family. Plant Physiology and Biochemistry 77: 108–116. 10.1016/j.plaphy.2014.02.002 24583344

[39] Liu X, Zhu J, Wei C, Guo Q, Bian C, Xiang Z, et al (2015) Genome-wide identification and characterization of the DREB transcription factor gene family in mulberry. Biologia Plantarum 59: 253–265.

[40] Larkin MA, Blackshields G, Brown NP, Chenna R, McGettigan PA, McWilliam H, et al (2007) Clustal W and Clustal X version 2.0. Bioinformatics 23: 2947–2948. 17846036 10.1093/bioinformatics/btm404

[41] Nicholas KB, Nicholas H, Deerfield D (1996) GeneDoc: analysis and visualization of genetic variation. Embnew News 4: 14.

[42] Bailey TL, Boden M, Buske FA, Frith M, Grant CE, Clementi L, et al (2009) MEME SUITE: tools for motif discovery and searching. Nucleic Acids Research 37: W202–W208. 10.1093/nar/gkp335 19458158 PMC2703892

[43] Liu L, Zhu K, Yang Y, Wu J, Chen F, Yu D (2008) Molecular cloning, expression profiling and trans-activation property studies of a DREB2-like gene from chrysanthemum (Dendranthema vestitum). Journal of Plant Research 121: 215–226. 10.1007/s10265-007-0140-x 18224275

[44] Chen H, Nelson R, Sherwood J (1994) Enhanced recovery of transformants of Agrobacterium tumefaciens after freeze-thaw transformation and drug selection. Biotechniques 16: 664–668, 670 8024787

[45] Clough SJ, Bent AF (1998) Floral dip: a simplified method for *Agrobacterium*-mediated transformation of *Arabidopsis thaliana* . The Plant Journal 16: 735–743. 10069079 10.1046/j.1365-313x.1998.00343.x

[46] Lodhi MA, Ye G-N, Weeden NF, Reisch BI (1994) A simple and efficient method for DNA extraction from grapevine cultivars and *Vitis* species. Plant Molecular Biology Reporter 12: 6–13.

[47] Chen P, Wang C, Li K, Chang J, Wang Y, Yang G, et al (2008) Cloning, expression and characterization of novel avenin-like genes in wheat and related species. Journal of Cereal Science 48: 734–740.

[48] Rae L, Lao NT, Kavanagh TA (2011) Regulation of multiple aquaporin genes in *Arabidopsis* by a pair of recently duplicated DREB transcription factors. Planta 234: 429–444. 10.1007/s00425-011-1414-z 21509693

[49] Voelker T, Sturm A, Chrispeels MJ (1987) Differences in expression between two seed lectin alleles obtained from normal and lectin-deficient beans are maintained in transgenic tobacco. The EMBO Journal 6: 3571 16453809 10.1002/j.1460-2075.1987.tb02687.x PMC553823

[50] Barrs H, Weatherley P (1962) A re-examination of the relative turgidity technique for estimating water deficits in leaves. Australian Journal of Biological Sciences 15: 413–428.

[51] Bates L, Waldren R, Teare I (1973) Rapid determination of free proline for water-stress studies. Plant and Soil 39: 205–207.

[52] Hodges DM, DeLong JM, Forney CF, Prange RK (1999) Improving the thiobarbituric acid-reactive-substances assay for estimating lipid peroxidation in plant tissues containing anthocyanin and other interfering compounds. Planta 207: 604–611. 10.1007/s00425-017-2699-3 28456836

[53] Chen H, Je J, Song C, Hwang JE, Lim CO (2012) A proximal promoter region of *Arabidopsis* DREB2C confers tissue-specific expression under heat stress. Journal of Integrative Plant Biology 54: 640–651. 10.1111/j.1744-7909.2012.01137.x 22716647

[54] Bhatnagar-Mathur P, Vadez V, Sharma KK (2008) Transgenic approaches for abiotic stress tolerance in plants: retrospect and prospects. Plant Cell Reports 27: 411–424. 18026957 10.1007/s00299-007-0474-9

[55] Tong Z, Hong B, Yang Y, Li Q, Ma N, Ma C, et al (2009) Overexpression of two *Chrysanthemum DgDREB1* group genes causing delayed flowering or dwarfism in *Arabidopsis* . Plant Molecular Biology 71: 115–129. 10.1007/s11103-009-9513-y 19544047

[56] Wang C, Deng P, Chen L, Wang X, Ma H, Hu W, et al (2013) A wheat WRKY transcription factor TaWRKY10 confers tolerance to multiple abiotic stresses in transgenic tobacco. PloS One 8: e65120 10.1371/journal.pone.0065120 23762295 PMC3677898

[57] Vinocur B, Altman A (2005) Recent advances in engineering plant tolerance to abiotic stress: achievements and limitations. Current Opinion in Biotechnology 16: 123–132. 15831376 10.1016/j.copbio.2005.02.001

[58] Bais HP, Ravishankar G (2002) Role of polyamines in the ontogeny of plants and their biotechnological applications. Plant Cell, Tissue and Organ Culture 69: 1–34.

[59] Liu J, Zhu JK (1997) Proline accumulation and salt-stress-induced gene expression in a salt-hypersensitive mutant of *Arabidopsis* . Plant Physiology 114: 591–596. 9193091 10.1104/pp.114.2.591 PMC158341

[60] Sathiyaraj G, Lee OR, Parvin S, Khorolragchaa A, Kim YJ, Yang DC. (2011) Transcript profiling of antioxidant genes during biotic and abiotic stresses in *Panax ginseng CA Meyer* . Molecular Biology Reports 38: 2761–2769. 10.1007/s11033-010-0421-7 21086178

