# Supplementary figures and images for: Mulberry Transcription Factor MnDREB4A Confers Tolerance to Multiple Abiotic Stresses in Transgenic Tobacco
Source: PLoS One. 2015 Dec 22;10(12):e0145619. doi: 10.1371/journal.pone.0145619 (PMC4687919; doi:10.1371/journal.pone.0145619)

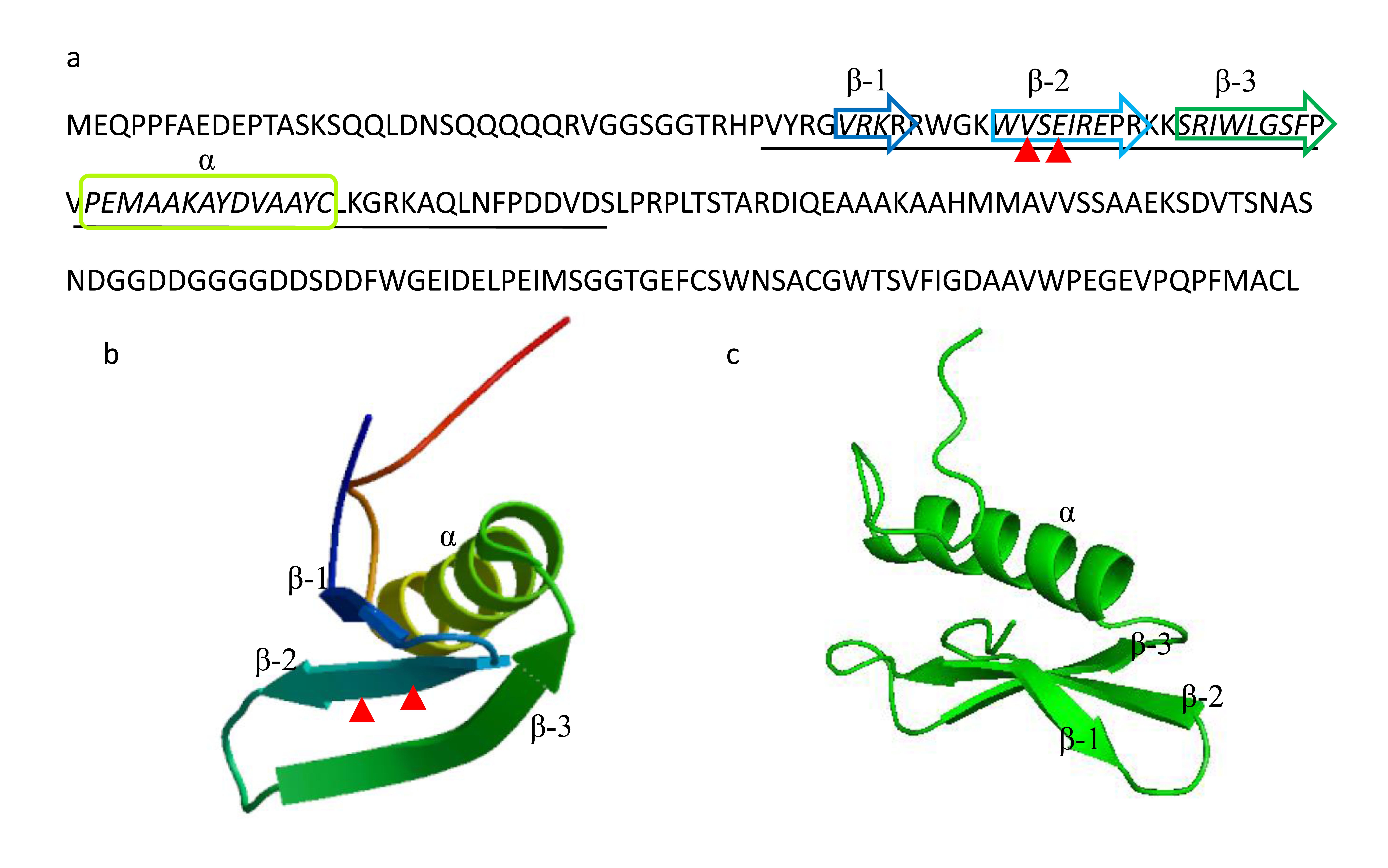

Supplement: S1 Fig — (a) The protein sequence of MnDREB4A. AP2 domains are highlight by the lines. Three β-folded sheets (β-1, β-2, and β-3) and one α-helix (α) were highlight by colorful arrows and rectangle. (b) The three-dimensional structure of MnDREB4A protein. The V14 and E19 were highlight by triangles. (c) The protein model was rotated a certain angles to show the structure. (JPG) [file pone.0145619.s001.jpg]

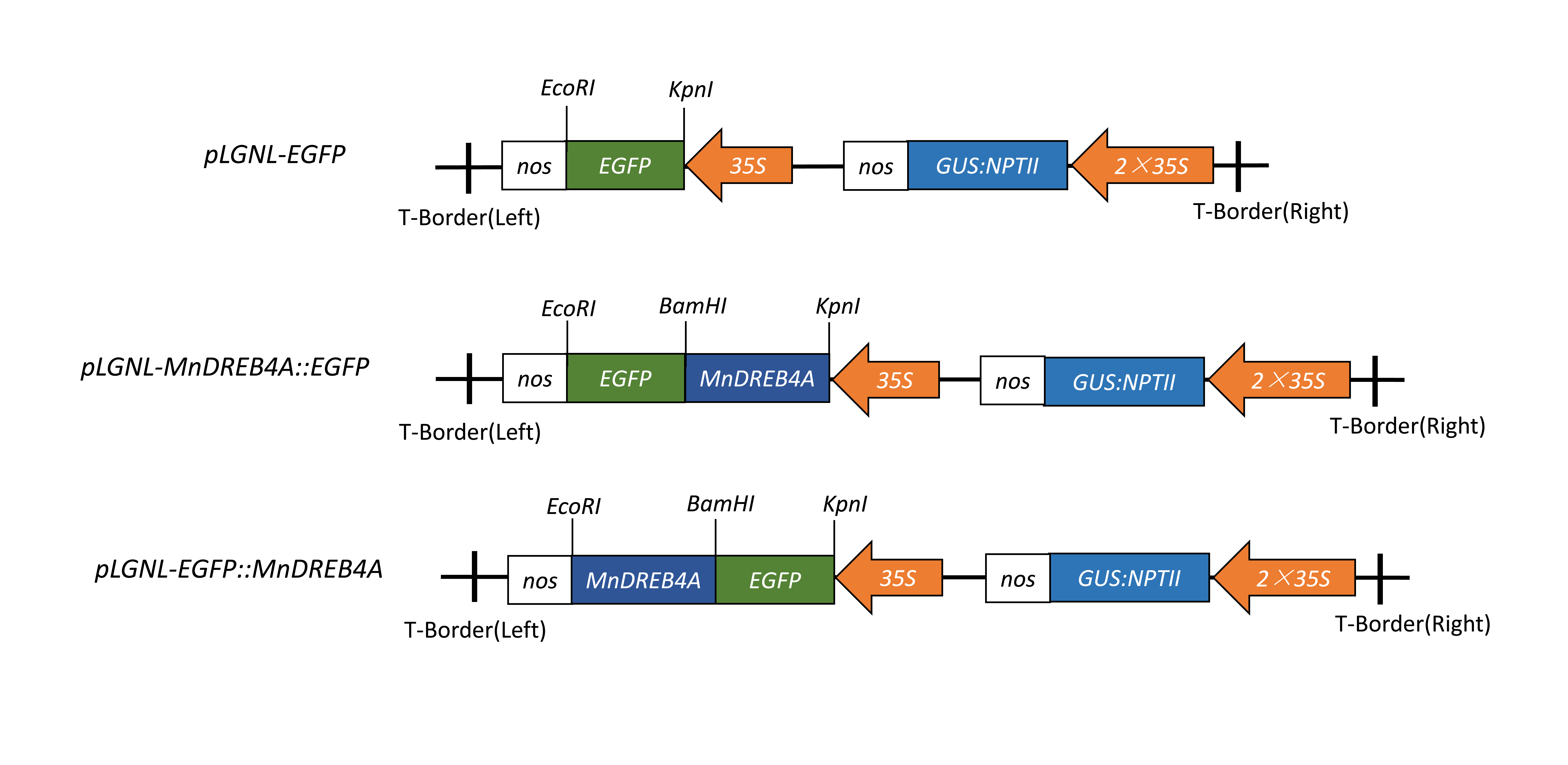

Supplement: S2 Fig — (JPG) [file pone.0145619.s002.jpg]

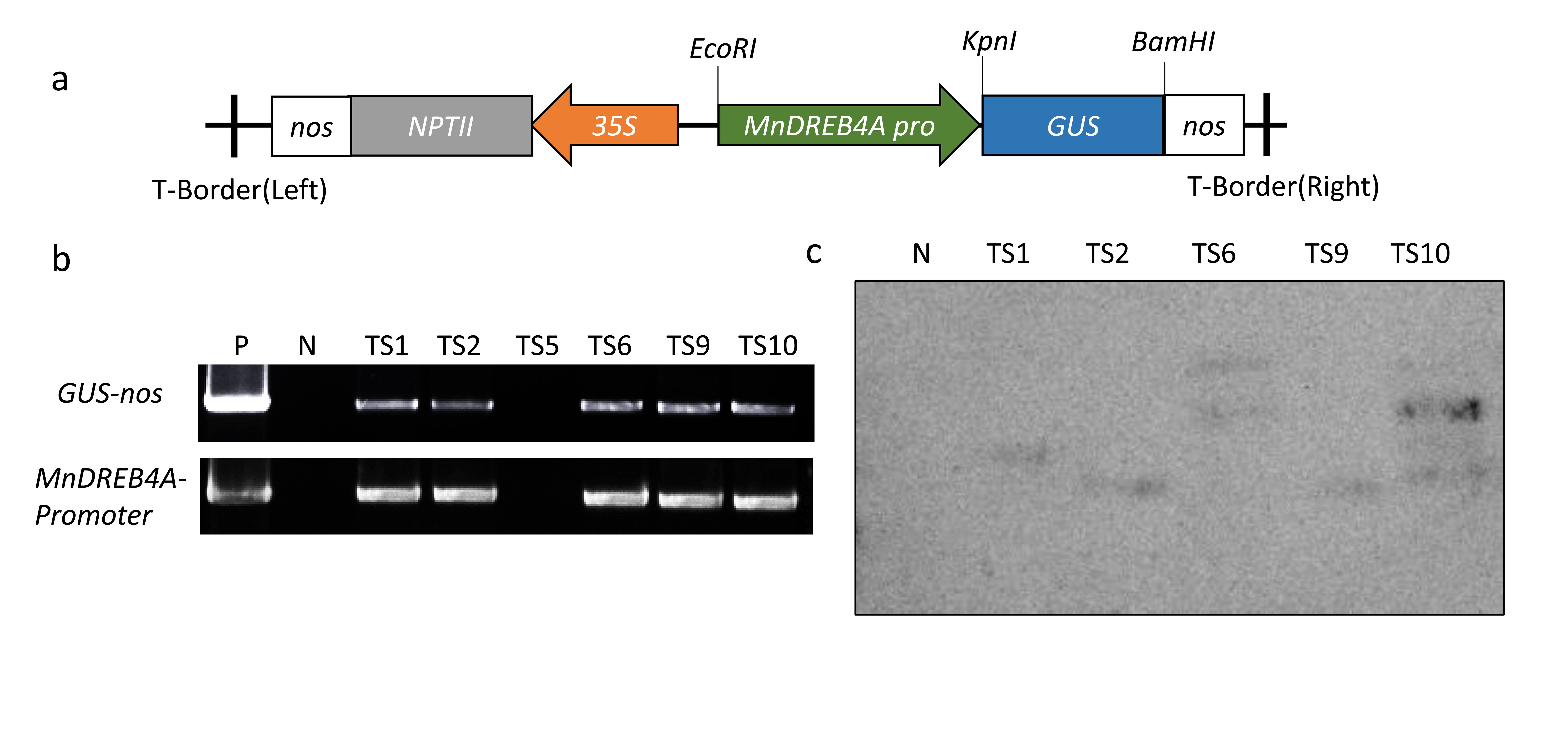

Supplement: S3 Fig — (a) The recombinant plasmid (MnDREB4A pro::GUS). (b) The transgenic lines were confirmed by genomic PCR. (c) Southern blot analysis of transgenic Arabidopsis. The recombinant plasmid was used as a positive control, and the genomic DNA of wild type (WT) Arabidopsis was used as negative control. P, N, and TSs indicate positive control, negative control, and transgenic lines, respectively. (JPG) [file pone.0145619.s003.jpg]

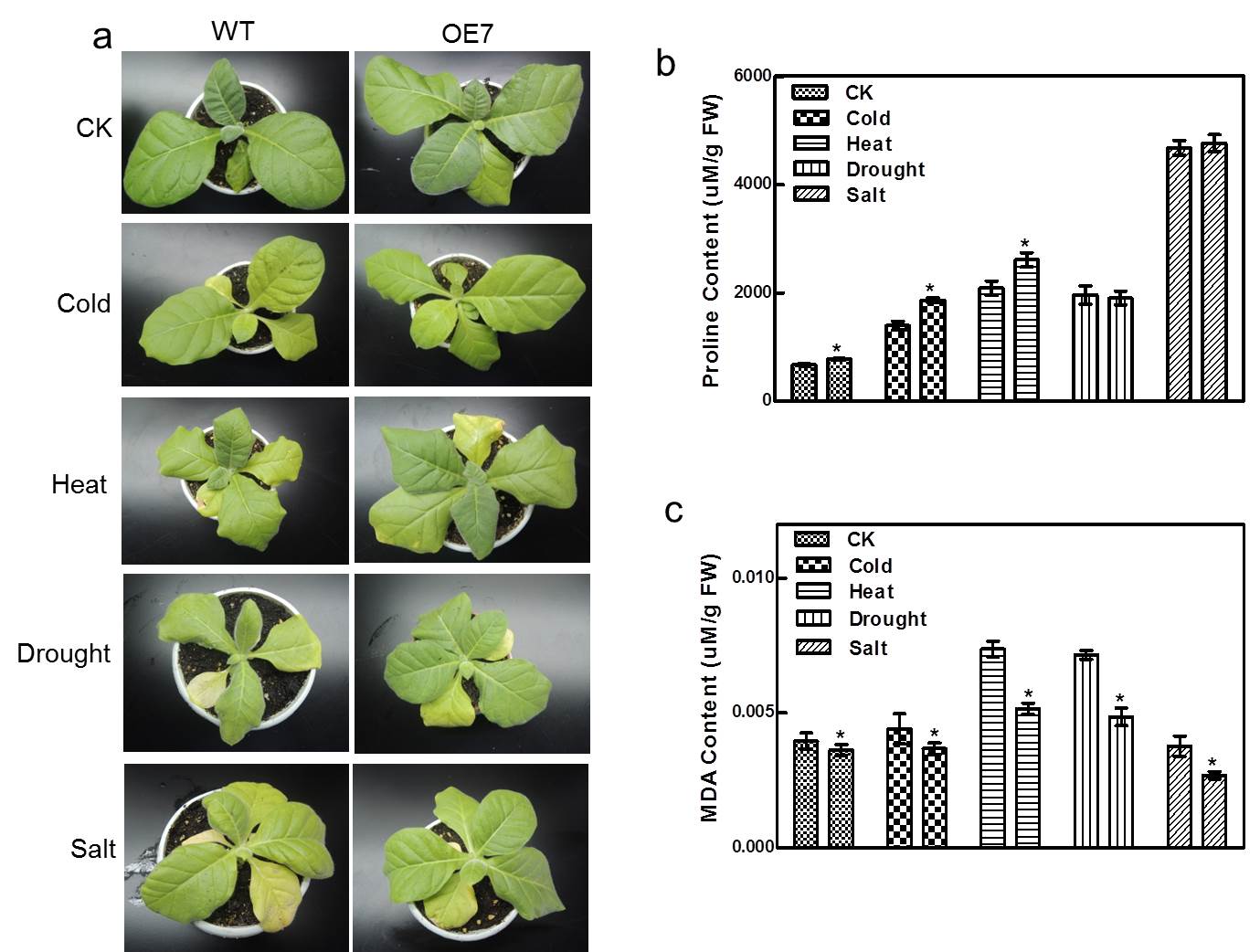

Supplement: S5 Fig — (a) The seedlings of transgenic OE7 and the WT (CK) were exposed to cold (4°C), heat (40°C), drought (20% PEG6000) and salt (400 mM NaCl). (b) The proline content after the abiotic stresses treatments. (c) The MDA content after the abiotic stresses treatments. (JPG) [file pone.0145619.s005.jpg]
