# Supplementary material for: Mulberry Transcription Factor MnDREB4A Confers Tolerance to Multiple Abiotic Stresses in Transgenic Tobacco
Source: PLoS One. 2015 Dec 22;10(12):e0145619. doi: 10.1371/journal.pone.0145619 (PMC4687919; doi:10.1371/journal.pone.0145619)
Supplement: S1 Table — (DOCX) [file pone.0145619.s007.docx]

**S1 Table. The DREB proteins used for protein sequence multi-alignment.**

| Species | Accession No or Gene ID |
| --- | --- |
| *Morus notabilis* | KF678389 |
| *Populus trichocarpa* | Potri.014G099900.1 |
| *Vitis vinifera* | VIT_15s0046g00310 |
| *Malus domestica* | MDP0000790788 |
| *Gossypium hirsutum* | DQ224383 |
| *Arabidopsis thaliana* | AED91698.1 |
| *Zea mays* | AAM80485.1 |
| *Oryza sativa* | LOC_Os04g46410 |
