# Supplementary material for: Mulberry Transcription Factor MnDREB4A Confers Tolerance to Multiple Abiotic Stresses in Transgenic Tobacco
Source: PLoS One. 2015 Dec 22;10(12):e0145619. doi: 10.1371/journal.pone.0145619 (PMC4687919; doi:10.1371/journal.pone.0145619)
Supplement: S2 Table — (DOCX) [file pone.0145619.s008.docx]

**S2 Table.** **List of primers used for constructing recombinant plasmids.**

| Vector | Gene | Primers（F/R） | Restriction enzyme sites | |
| --- | --- | --- | --- | --- |
| *EGFP::MnDREB4A* | *EGFP* | 5’-GGGGTACCATGGTGAGCAAGGGCGA-3’ | | *Kpn* I |
|  |  | 5’-CGGGATCCCTTGTACAGCTCGTCCATGC-3’ | | *Bam*H I |
|  | *MnDREB4A* | 5’-CGGGATCCGGAGGAGGAGGATCAGGAGGAGGAGGAT CAGGAGGAGGAGGATCAATGGAGCAACCACCATTC-3’ | | *Bam*H I |
|  |  | 5’-CGGAATTCCTAGAGGCACGCCATGAA-3’ | | *Eco*R I |
| *MnDREB4A::EGFP* | *EGFP* | 5’-CGGGATCCATGGTGAGCAAGGGCGA-3’ | | *Bam*H I |
|  |  | 5’-CGGAATTCTTACTTGTACAGCTCGTCCATG-3’ | | *Eco*R I |
|  | *MnDREB4A* | 5’-GGGGTACCATGGAGCAACCACCATTCG-3’ | | *Kpn* I |
|  |  | 5’-CGGGATCCTGATCCTCCTCCTCCTGATCCTCCTCCTCCTGATCCTCCTCCTCCGAGGCACGCCATGAACG-3’ | | *Bam*H I |
| *MnDREB4A pro:: GUS* | *MnDREB4A-promoter* | 5’-CGGAATTCGTTTACTTGAAGGGCCTAA-3’ | | *Eco*R I |
|  |  | 5’-ATGGTACCTGCCAAATGCCAACTATTAC-3’ | | *Kpn* I |
|  | *GUS-nos* | 5’-GGGGTACCATGTTACGTCCTGTAGAAA-3’ | | *Kpn* I |
|  |  | 5’-CGGGATCCCCCGATCTAGTAACATAG-3’ | | *Bam*H I |
| *CaMV35S::MnDREB4A* | *MnDREB4A* | 5’-GCGGTACCATGGAGCAACCACCA-3’ | | *Kpn* I |
|  |  | 5’-GGACTAGTCTAGAGGCACGCCATG-3’ | | *Spe* I |
